# ASO Author Reflections: Prediction of the Chemotherapy Effect on the Prognosis of Locally Advanced Pancreatic Cancer

**DOI:** 10.1245/s10434-022-12579-w

**Published:** 2022-10-08

**Authors:** Masayuki Tanaka, Thilo Hackert

**Affiliations:** 1 Department of General, Visceral and Transplantation Surgery, University of Heidelberg, Heidelberg, Germany; 2 Department of Surgery, Keio University School of Medicine, Keio, Japan

## Past

The use of potent multiagent chemotherapy protocols has resulted in increased opportunities for potentially curative resection, including resection for locally advanced pancreatic cancer (LAPC).^1,2^ However, identifying the patients who would benefit from conversion surgery for LAPC has been a matter of debate during the past decade.^3,4^ Evidence-based guidelines for conversion surgery after induction chemotherapy are currently lacking. This study therefore focused on the preoperative factors predictive of overall survival for patients with resected LAPC after induction FOLFILINOX and established a prognostic model with these factors that may guide optimal treatment for LAPC.^5^

## Present

In the current study, the patients who fulfilled the following criteria after FOLFIRINOX for LAPC underwent surgery: no tumor progression on imaging, declining (or stable) carbohydrate antigen 19-9 (CA19-9) values, good general condition, and technically resectable disease.^5^ If no progressive disease was detected intraoperatively, patients underwent oncologic resection of the primary pancreatic tumor. In this context of patient selection for surgical resection after induction therapy, tumor shrinkage and density on computed tomography (CT) and post-chemotherapy CA19-9 serum levels were recognized as predictive factors for the prognosis. With these three factors, a scoring system for overall survival was established. Patients with a higher score are definitive candidates for resection and may achieve excellent prognosis. However, patients with a low score may not necessarily have to be excluded from surgical exploration, but may be candidates for additional treatment.

## Future

Some patients experienced favorable radiologic and biologic responses to induction FOLFIRINOX, as assessed by cross-sectional imaging and CA 19-9. Although the scoring system developed in this study might be helpful for treatment decisions, it is uncertain whether this model is applicable to all patients satisfying different criteria for conversion surgery or treated with different chemotherapy regimens. The true impact of the patient selection and the score model on decision-making for the conversion requires re-evaluation in further studies, preferably within a multicenter prospective study design. One additional concern is the application of an optimal biomarker that is highly sensitive to tumor biology in the setting of induction chemotherapy for LAPC.
